# The importance of alcohol abuse and smoking in the evolution of glaucoma disease

**Published:** 2013-06-25

**Authors:** SM Chiotoroiu, D Pop de Popa, GI Ştefăniu, FA Secureanu, VL Purcărea

**Affiliations:** * “Nicolae Malaxa" Clinical Hospital, Bucharest; ** “Carol Davila" University of Medicine and Pharmacy, Bucharest; *** Clinical Ophthalmological Emergency Hospital

**Keywords:** glaucoma disease, alcohol abuse, smoking

## Abstract

Background:Diagnosing glaucoma by clinical and paraclinical selection, monitoring the patients who present glaucoma, smoke and consume high quantities of alcohol. We wish to demonstrate the influence of smoking and alcohol consumption on the progression of glaucoma.

Material and Method: The paper represents a clinical prospective observational and interventional study within a period of 11 months (September 2011- August 2012), which includes 214 patients diagnosed with glaucoma, by clinical and paraclinical examination, and, who were administrated prostaglandin analogues. The group was divided into 4 homogeneous subgroups according to age, without ocular and systemic associated pathology: Group A- patients diagnosed with glaucoma who do not smoke or drink alcohol (witness group). Group B- patients diagnosed with glaucoma who smoke but do not drink alcohol. Group C- patients diagnosed with glaucoma who drink alcohol but do not smoke. Group D- patients diagnosed with glaucoma who smoke and drink alcohol.

The patients in the 4 groups were supervised by monthly periodical examinations in the first 3 months, then at 3 months by clinical examination (anterior pole examination, eye background) and paraclinical examination (gonioscopy, aplanotonometry Goldman, pachymetry, retinography, computerized perimetry, optical coherence tomography - OCT).

Results:Tensional variations in all the 4 groups under treatment were between (+2;-2mmHg). Retinography failed to point out significant changes from the enrolment to the moment of the paper presentation. The visual field has registered important changes in groups B and D and has not registered significant changes in groups A and C. OCT changes were registered particularly in group D.

Conclusions:The progression of the glaucoma disease is more influenced by smoking than by alcohol consumption. The association between smoking and alcohol consumption certainly represents an agent of major risk in the progression of glaucoma.

## Introduction

Glaucoma is a bilateral, chronic, progressive and multifactorial optical neuropathy characterized by the death of ganglion retinal cells. Cells’ death appears by apoptosis (programmed death), caused in unknown conditions with the contribution of some risk factors: (i) growth of intra ocular pressure (IOP), (ii) genetic factors, (iii) vascular factors, (iv) toxics. 

The theories involved in glaucoma ethiopathology: (a) mechanical theory - the compression of the ganglion retinal cells at the level of cribrosa lamina due to IOP growth, which determines the blockage of axonal retrograde flow, that brings to the perikaryon the neurotrophic factors produced at the level of lateral geniculate bodies; (b) the vascular theory - the decrease of the retinal vascular flow, the loss of ganglion retinal cells [P = (Pa-Pv) /R] (P- perfusion pressure, Pa- arterial pressure, Pv- venous pressure, R- flow resistance). Any growth of Pv or decrease of Pa determines a decrease of perfusion pressure. Young people present autoregulation factors like endothelin or nitric oxide, which decrease R. 

**Work assumptions**

 Smoking and alcohol abuse are factors involved in the appearance of optical alcohol-tobacco neuropathy (alcohol-tobacco amblyopia). The mechanism of appearance is represented by dysfunctions at the mitochondrial level. Mitochondria is known as the main player in the cells’ survival or death especially of neurons; the existence of ganglion retinal cells and their axons depends on mitochondrial energy. The mitochondria’s biogenesis is the result of a sort of coordinating interaction between the nuclear and mitochondrial genome. These mechanisms are involved in optical neuropathy (Leber, vitamins’ deficiency, nutritional deficiencies, and the use of certain drugs). 

 Among the numerous drugs and agents involved with glaucoma by diverse mechanisms is tobacco emerges. Smoking is associated with an immediate rise of IOP by vasoconstriction, which determines the rise of pressure in the episcleral veins; consequently, the reduction of aqueous humor outflow and the rise of IOP appear. People with GPUD who stop smoking for a month display a decrease of IOP with 2-5 mmHg. (Wilson and coworkers showed a direct relation between smoking and glaucoma). Shephard and coworkers claim that there is no relation between smoking, IOP growth and glaucoma. 

** Mechanisms through which alcohol determines the decrease of IOP **

 Previous studies showed that alcohol determines an important decrease of IOP in glaucomatous eyes and minimum changes in normal patients if approx. 50 c.c. of alcohol are consumed. In glaucoma, the characteristic response at this dose is a decrease of IOP in the first hour, effect maintained for over 2-3 hours (Rouland E. and W. Marton). Changes of blood osmotic pressure induce changes of IOP: (a) the growth of IOP is induced by hypoosmolarity (ex: test of water ingestion); (b) the decrease of IOP is induced by hyperosmolarity (urea, mannitol, glycerol, ethanol). Glaucomatous eyes are more sensitive in osmolarity variations that normal eyes probably have a drain decrease. Alcohol stimulates diuresis by the suppression of the circulating antidiuretic hormone. The hormone can directly influence water circulation in the eyes as well as in the kidneys. As far as the effect of antidiuretic hormone is concerned, it is known to activate on output and circulation of the aqueous humor little. 

The aim is to diagnose glaucoma, to monitor the patients who present glaucoma, smoke and consume high quantities of alcohol.

## Materials and methods

The paper represents a clinical prospective observational and interventional study within a period of 4 years. These are the preliminary results from the first 12 months (September 2011- August 2012). We included 114 patients diagnosed with glaucoma by clinical and paraclinical examinations, who were administered prostaglandin analogues. The group was divided into 4 homogeneous subgroups according to age, without ocular and systemic associated pathology: Group A - 20 patients diagnosed with glaucoma, who did not smoke or drink alcohol (witness group); Group B - 35 patients diagnosed with glaucoma who smoked but did not drink alcohol; Group C - 28 patients diagnosed with glaucoma who drank alcohol but did not smoke; Group D - 31 patients diagnosed with glaucoma who smoked and drank alcohol. The patients from the 4 groups were supervised by monthly periodical examinations in the first 3 months, then at 6 months by clinical examination (anterior and posterior pole examination) and paraclinical examination (gonioscopy, Pascal tonometry, pachymetry, retinophotography, visual fields, OCT). 

**Fig. 1:**
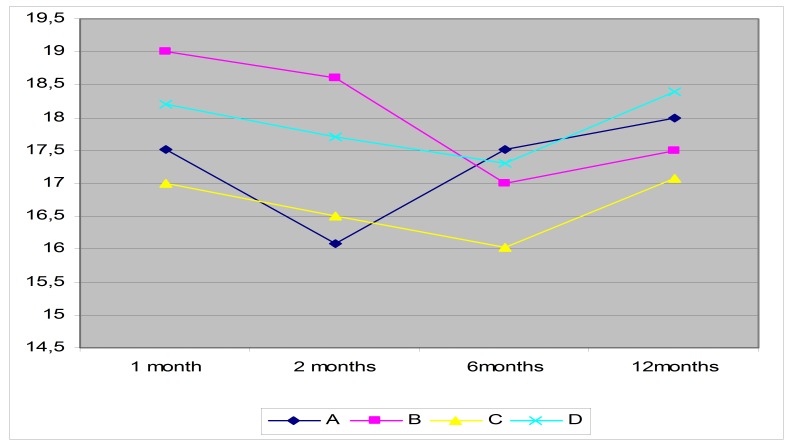
The evolution of the average IOP of the four groups during the study

**Fig. 2:**
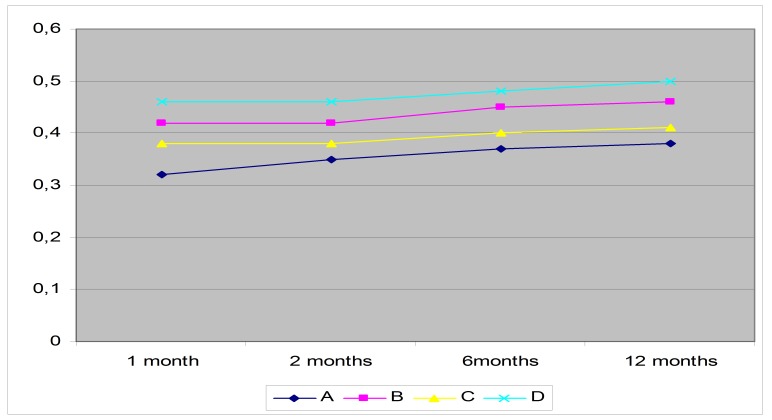
The evolution of the average values of C/D in the four groups during the study

The mean ratio cup/ disc (C/D) examined by retinal photography and OCT was between 0,3 and 0,5 with higher values in groups B and D (**Fig. 2**).

**Fig. 3:**
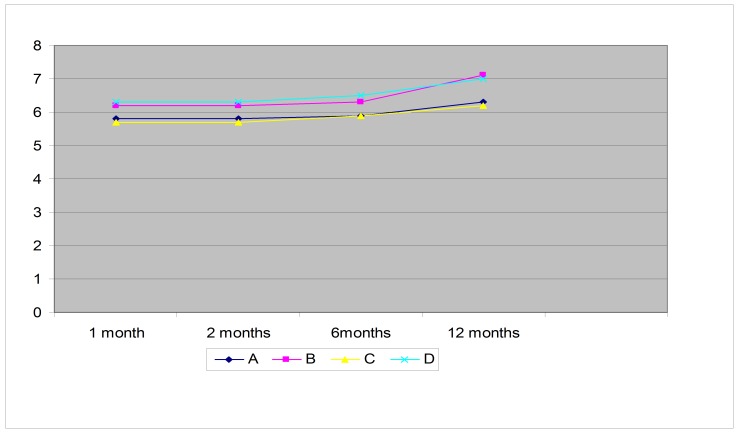
The evolution of the average values of MD during the study showed by visual field (Humphrey 24-2).

 Values of the medium deviation (MD) were between 5,5 and 7 during the study, with higher values in groups B and D (**Fig. 3**).

## Discussions

It is known that glaucoma is a major cause of irreversible blindness worldwide. Its cause remains unknown, so the study of the risk factors for this optic neuropathy is pivotal for the management of the disease and the avoidance of the progression to blindness [**1**]. Thus, we have studied the influence of smoking on the progression of this disease, taking into consideration that the eye is naturally exposed to the environment, which means that cigarette smoke can directly affect it [**2**]. Grzybowski has shown that smoking produces ischemia and oxidative stress and that smoking has a negative impact on glaucoma surgery [**4**]. Cheng et al. [**15**] also found a strong association between smoking and glaucoma, suggesting that the damage from smoke is probably due to the presence of toxic substances that induce an increase in free radicals and a decrease in antioxidants [6,10]. Cigarette smoking increases the risk of vascular disease [**7**]. Glaucoma interferes with the blood flow to the optic nerve head and thus may have a vascular origin [**16**]. A case-control study found that current cigarette smoking was related to glaucoma presence in multiple logistic regression analysis [**14,18**]. Other studies failed to find an association between smoking and glaucoma [**12,13**]. Specifically, smoking was not related to open-angle glaucoma in a population-based cross-sectional study of Hispanics aged older than 40 years, residing in Arizona [**11**]. Previous or current smoking was not associated with the presence of glaucoma in the population-based study conducted in Beaver Dam, Wisconsin, in individuals ages 43 to 84 [**9**]. In two case-control studies, one conducted in Congo [**8**], and another in France [**5**], researchers found smoking was not related to glaucoma status. In a recent meta-analysis of existing studies, an association existed between having primary open-angle glaucoma and current smoking; no relationship with former smoking was present [**3**]. Smoking status has a strong association with eye health, especially age-related macular degeneration, but population-based studies found no association in the area of glaucoma [**17**].

## Conclusion

Tensional variations in all the 4 groups under treatment were between (+2;-2 mmHg). Retinal photography has not pointed out significant changes from the enrolment to the moment of the paper presentation. The visual field has registered changes in groups B and D and has not registered significant changes in groups A and C. OCT has not registered any changes. The progression of the glaucoma disease is more influenced by smoking then by alcohol consumption. The association between smoking and alcohol consumption certainly represents a major risk in the progression of glaucoma.
